# Renal and Cardiovascular Effects of SGLT2 Inhibition in Combination With Loop Diuretics in Patients With Type 2 Diabetes and Chronic Heart Failure: The RECEDE-CHF Trial

**DOI:** 10.1161/CIRCULATIONAHA.120.048739

**Published:** 2020-08-29

**Authors:** Natalie A. Mordi, Ify R. Mordi, Jagdeep S. Singh, Rory J. McCrimmon, Allan D. Struthers, Chim C. Lang

**Affiliations:** 1 Division of Molecular and Clinical Medicine, University of Dundee, Scotland, United Kingdom (N.A.M., I.R.M., R.J.M., A.D.S., C.C.L.).; 2 Royal Infirmary of Edinburgh, Scotland, United Kingdom (J.D.S.).

**Keywords:** diabetes mellitus, diuretics, furosemide, heart failure

## Abstract

**Background:**

SGLT2 (sodium-glucose cotransporter-2) inhibitors improve heart failure–associated outcomes in patients with type 2 diabetes. In patients with heart failure, SGLT2 inhibitors will likely be coprescribed with a loop diuretic, but this combined effect is not well-defined. Our aim was to assess the diuretic and natriuretic effect of empagliflozin in combination with loop diuretics.

**Methods:**

The RECEDE-CHF trial (SGLT2 Inhibition in Combination With Diuretics in Heart Failure) was a randomized, double-blind, placebo-controlled, crossover trial of patients with type 2 diabetes and heart failure with reduced ejection fraction taking regular loop diuretic who were randomized to empagliflozin 25 mg once daily or placebo for 6 weeks with a 2-week washout period. The primary outcome was change in 24-hour urinary volume from baseline to week 6.

**Results:**

Twenty-three participants (mean age, 69.8 years; 73.9% male; mean furosemide dose, 49.6±31.3 mg/d; mean HbA1c, 7.9±3.8%) were recruited. Compared with placebo, empagliflozin caused a significant increase in 24-hour urinary volume at both day 3 (mean difference, 535 mL [95% CI, 133–936]; *P*=0.005) and week 6 (mean difference, 545 mL [95% CI, 136–954]; *P*=0.005) after adjustment for treatment order, baseline 24-hour urine volume, and percentage change in loop diuretic dose. At 6 weeks, empagliflozin did not cause a significant change in 24-hour urinary sodium (mean difference, −7.85 mmol/L [95% CI, −2.43 to 6.73]; *P*=0.57). Empagliflozin caused a nonsignificant increase in fractional excretion of sodium at day 3, which was absent at week 6 (mean difference day 3, 0.30% [95% CI, −0.03 to 0.63]; *P*=0.09; week 6, 0.11% [95% CI, −0.22 to 0.44]; *P*>0.99), and a significant increase in electrolyte-free water clearance at week 6 (mean difference, 312 mL [95% CI, 26–598]; *P*=0.026) compared with placebo. Empagliflozin also caused significant reductions in body weight and serum urate at week 6.

**Conclusions:**

Empagliflozin caused a significant increase in 24-hour urine volume without an increase in urinary sodium when used in combination with loop diuretic.

**Registration:**

URL: https://www.clinicaltrials.gov; Unique Identifier: NCT03226457.

## Abstract 2

Supplemental Digital Content is available in the text.

**Clinical Perspective**

**What Is New?**

- In patients with heart failure and type 2 diabetes taking regular loop diuretics, empagliflozin caused a significant increase in urine volume at both day 3 and week 6 compared with placebo, as well as a significant increase in electrolyte free water clearance.
- Although there was small, nonsignificant increase in natriuresis with empagliflozin at day 3, this was absent by week 6.

**What Are the Clinical Implications?**

- These results suggest empagliflozin may have an advantageous diuretic profile in patients with type 2 diabetes and heart failure in addition to loop diuretics, with only a short, transient natriuresis.

SGLT2 (sodium-glucose cotransporter-2) inhibitors have demonstrated improved cardiovascular and renal outcomes in patients with type 2 diabetes (T2D), most strikingly with a significant reduction in hospitalization for heart failure (HF).^1–3^ Recently, the SGLT2 inhibitor dapagliflozin has been shown to cause a reduction in death and HF hospitalization in patients with HF with reduced ejection fraction irrespective of T2D status.^4^ One notable feature from these outcome trials was the evidence of early benefit (<3 months). In DAPA-HF (Study to Evaluate the Effect of Dapagliflozin on the Incidence of Worsening Heart Failure or Cardiovascular Death in Patients With Chronic Heart Failure), the reduction in worsening HF events, defined as either an unplanned hospitalization or an urgent visit requiring intravenous therapy for HF, was seen as early as the first few weeks.^4^ The mechanism for this is unclear, but one possible hypothesis is the diuretic effect of SGLT2 inhibition.^5–8^

The SGLT2 is localized to the renal proximal convoluted tubules, acting to reabsorb the majority (≈90%) of the filtered glucose coupled with sodium. SGLT2 inhibition therefore results in glucosuria, and the ensuing osmotic diuresis may potentially be beneficial particularly for those with T2D and HF.^6,9^ Whether SGLT2 inhibitors cause significant natriuresis is less clear, but is important in the context of patients with HF who are likely to also be prescribed loop diuretics.

Given the improvement in HF-associated outcomes seen with SGLT2 inhibitors, the results of DAPA-HF, and the recent Food and Drug Administration approval for the use of dapagliflozin in patients with HF with reduced ejection fraction, the coprescription of loop diuretic and SGLT2 inhibitors will become increasingly common,^4,10^ and therefore further mechanistic studies are required to understand their combined effects. The aim of this trial was to explore the effects of the SGLT2 inhibitor empagliflozin on diuresis and natriuresis and on the interaction between loop diuretics and SGLT2 inhibitors.

## Methods

### Study Design

The study was approved by the East of Scotland Research Ethics Service (Regional Ethics Committee reference 16/ES/0137) and all patients gave written informed consent before study inclusion. All data generated or analyzed during this study are included in this published article and its supplementary information files.

**Figure 1.**
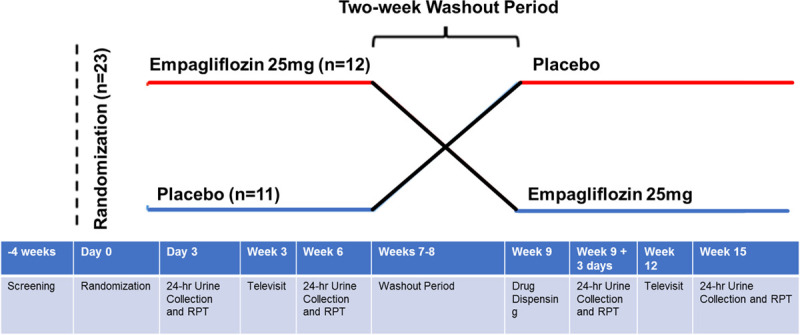
**The RECEDE-CHF (SGLT2 Inhibition in Combination With Diuretics in Heart Failure) study design.** Crossover design of the trial. RPT indicates renal physiologic test.

The full details of the RECEDE-CHF trial (SGLT2 Inhibition in Combination With Diuretics in Heart Failure) (NCT03226457) methodology have been published.^11^ In brief, RECEDE-CHF was a single-center, randomized, double-blind, placebo-controlled, crossover trial conducted in Tayside, Scotland, to compare the SGLT2 inhibitor empagliflozin with placebo. After successful screening for eligibility and safety, participants were randomized to either empagliflozin 25 mg/placebo or placebo/empagliflozin 25 mg for 6 weeks with a minimum of a 2-week washout period between treatment arms. Patients were randomized in a 1:1 fashion with group assignment masked by use of encapsulated study drug/placebo (Figure 1). Randomization was carried out by an independent pharmacist with no other trial role, using block randomization of treatment order using a validated randomization program. Both the investigator and the patient were blinded to the treatment order.

### Inclusion and Exclusion Criteria

Patients were eligible if they were 18 to 80 years of age with previously diagnosed T2D, with a documented diagnosis of HF, in New York Heart Association functional Class II or III, with previous echocardiographic evidence of HF with left ventricular ejection fraction <50%. HF was deemed to be the diagnosis when there was documentation from the treating clinician of presentation with symptoms or signs consistent with HF requiring treatment with loop diuretics in the presence of an echocardiogram confirming left ventricular ejection fraction <50%. Participants were required to be on stable doses of furosemide (or alternative loop diuretic) for at least 1 month before randomization and not have been hospitalized for HF for at least 3 months before giving consent.

Patients were excluded if they had systolic blood pressure <95 mm Hg at screening visit, had HbA1c <6.0%, had estimated glomerular filtration rate (eGFR) <45 mL/min/73 m^2^, were taking regular thiazide diuretics, or had a diagnosis of chronic liver disease or liver enzymes twice the upper limit of normal.

### Study Procedures

After the randomization visit, participants were given empagliflozin 25 mg or placebo. Participants returned at 3±2 days, having completed a 24-hour urine collection before this visit, for a study day where they underwent renal physiologic tests (RPTs). The RPTs were performed to provide further mechanistic understanding on the combined effect of administration of empagliflozin in addition to furosemide immediately after the dose. The specific aim of these RPTs was to study the acute effects of empagliflozin versus placebo in the first hour of administration on urine volume and urinary sodium excretion. The protocol was also designed to test whether empagliflozin augmented the effect of a bolus of intravenous loop diuretic. Full details of the RPTs are described in the previously published methods article^11^ and in Table I in the Data Supplement. In brief, the RPTs took place over ≈5 hours and followed an overhydration protocol. Participants took an oral water load (15 mL/kg) and voided urine at 30-minute intervals. After each void of urine, the patient drank a volume of water that was equal to the volume of urine voided to induce steady-state diuresis and avoid the need for catheterization. The participants received empagliflozin 25 mg or placebo at 150 minutes, with ongoing 30-minute urine collections. One hour later (210 minutes), they received a bolus of intravenous furosemide at half their total daily dose, and a further 2 urine volumes were measured at 30 minutes and 1 hour after injection. Intravenous furosemide was administered to eliminate variable gut absorption and for practical reasons to allow study completion within the set time frame owing to the reduced time for peak onset of diuretic effect with intravenous administration.^11^

Patients returned at week 6 having performed another 24-hour urine collection and underwent another RPT. The study drug, empagliflozin 25 mg or placebo, was discontinued at this point. The patient then returned after a minimum of a 2-week washout period (week 9) to begin the second treatment arm of placebo or empagliflozin 25 mg. The 24-hour urine collection and RPT was then repeated at week 9+3 days and at week 14 for the final study day, when all patients terminated the study drug.

### Study Outcomes

The primary outcome of the trial was to assess whether empagliflozin augmented the diuretic effects of loop diuretics as measured by the absolute change in 24-hour urine volume (mean difference), when compared with placebo, measured at week 6.

Secondary outcomes included change in 24-hour urinary sodium (mmol/L and mmol/d), fractional excretion of sodium (FENa), assessment of electrolyte-free water clearance (mL), changes in renal biomarkers (serum creatinine, eGFR, cystatin C, and urine protein/creatinine and albumin/creatinine ratios), and change in NT-proBNP (N-terminal pro–B-type natriuretic peptide).

FENa was calculated using the following formula:


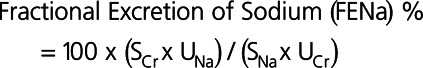


where S_Cr_ is the serum creatinine, U_Na_ is the urine sodium, S_Na_ is the serum sodium, and U_Cr_ is the urine creatinine.

The effect of empagliflozin versus placebo on urinary volume, urine sodium, and FENa during the RPTs was also assessed. Because of the large volume of multicenter studies reporting on the safety and adverse events of SGLT2 inhibitors, for this trial the focus was on the effects on coprescription with loop diuretic. Adverse event data were also collected.

### Statistical Analysis

We hypothesized that changes in urine output seen during the RPT would also be extrapolated over the 24-hour urine collection period, given the relative urine volume magnitudes. We therefore elected to base our sample size calculation on the effect of empagliflozin versus placebo in the RPT. A previous study in which an RPT was performed using a similar overhydration protocol reported a mean furosemide-induced urinary volume of 920 mL over 1 hour (SD 250 mL). We hypothesized that empagliflozin would cause an additional 20% increase in urinary volume. Twenty-two participants per arm were required to detect this difference with an α of 0.05 and power of 90%. This also gave 80% power (α=0.05) to detect a 20% increase in mean furosemide-induced sodium excretion of 300 µmol/min (SD 60).^12^ We decided that because the RPT days were likely to be highly intensive for the patients, we would also factor in a high dropout rate, and so we initially planned to recruit 34 patients.

Number and percentage were calculated for discrete variables. For continuous variables, mean and SD are reported where data are evenly distributed and median and interquartile range for non-normally distributed data.

Analyses were performed on the primary and secondary measures comparing empagliflozin versus placebo and assessed by 2-way analysis of covariance correcting for treatment order, baseline value, and any percentage change in furosemide dose at the visit. Data for continuous outcome measures were assessed for normality before analysis. Transformation of the outcome variables was used where necessary where these were not normally distributed. In analysis of the electrolyte free water clearance (cH20e), multiple imputations were used to impute missing values because there was >10% missing data. Ten imputations were carried out and pooled results calculated. Because 24-hour urine collections were not performed at the randomization visit (day 0), values taken from day 3 of placebo were taken as baseline. For the RPTs, urine volume, sodium, and FENa at the beginning of the RPT and after administration of study drug and furosemide were directly compared between empagliflozin and placebo at both day 3 and week 6.

## Results

### Baseline Characteristics

**Figure 2.**
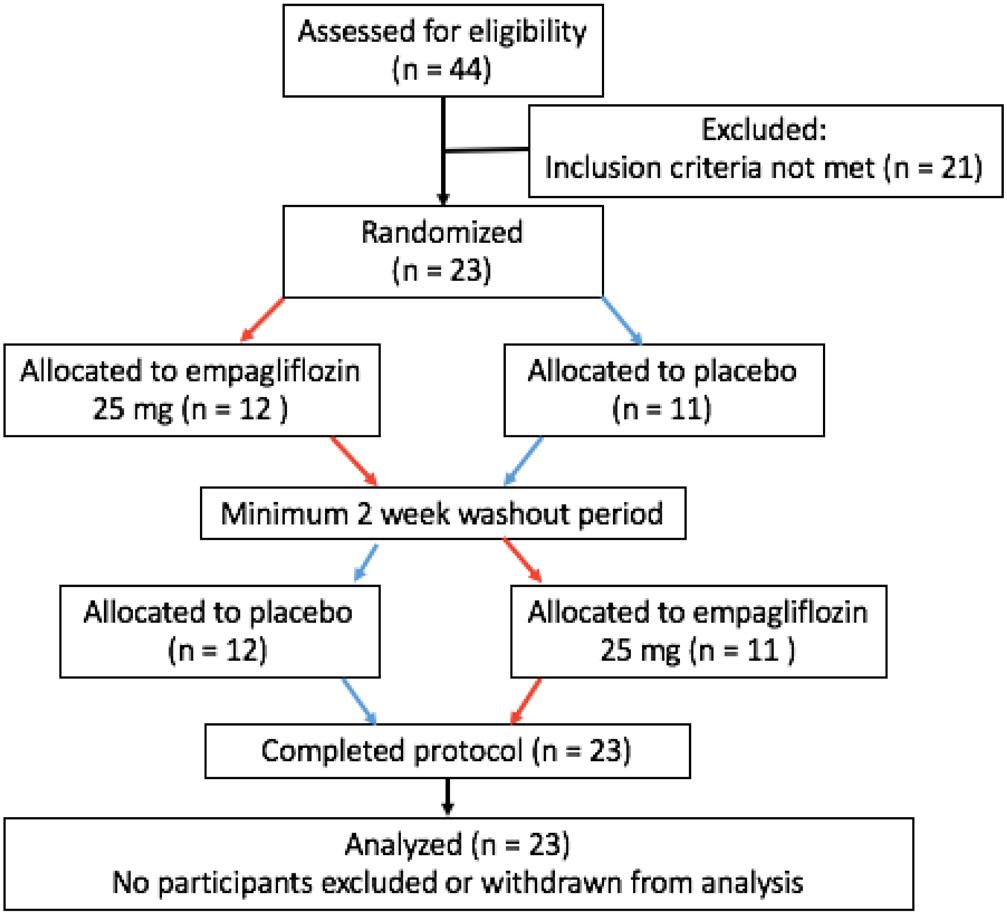
**Consolidated Standards of Reporting Trials diagram.** Screening, recruitment, and study completion.

Recruitment is summarized in the Consolidated *Standards* of Reporting Trials diagram (Figure 2) and baseline characteristics are summarized in Table 1.

**Table 1.**
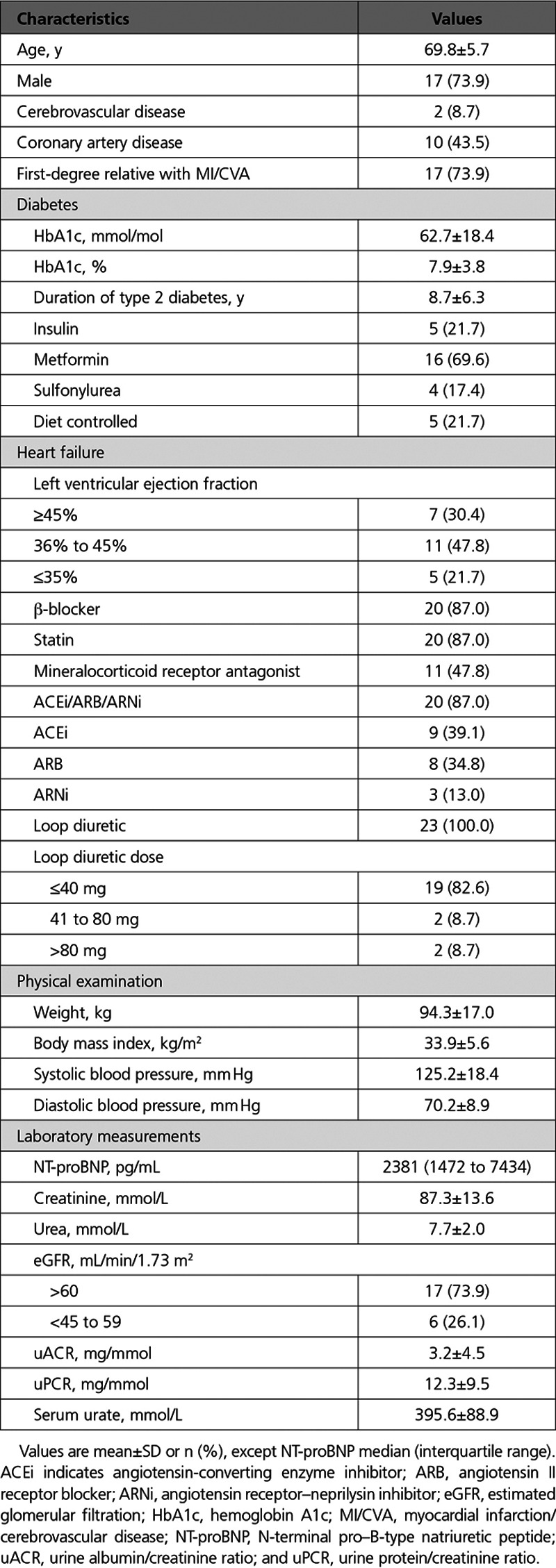
Baseline Data (n=23)

Between December 2017 and August 2018, 44 patients were screened for eligibility and 23 patients were randomized. The anticipated high dropout from the study did not occur, and all patients remained in the study for the duration; therefore, for ethical reasons, we stopped recruitment at 23 patients, once it became clear that we would have an adequate sample to meet our prespecified power calculation.

Of the 21 patients who were screen failures, 10 failed because of HbA1c <6.0%, 7 failed because of reduced eGFR, 1 patient was also on a thiazide diuretic, 1 had started on an SGLT2 inhibitor, 1 was not on stable HF therapy (this patient had recently started taking sacubitril/valsartan), and 1 was not able to be randomized within 4 weeks as per protocol.

The mean age of the cohort was 69.8±5.7 years and the majority of the patients were men (73.9%). As per the inclusion criteria, all patients were in New York Heart Association functional class II to III with previous echocardiographic evidence of HF with left ventricular ejection fraction <50% (47.8% had an ejection fraction of 36% to 45%), with a median NT-proBNP of 2381 pg/mL (range, 1472–7434). All participants were taking furosemide or equivalent loop diuretic at a mean dose of 49.6±31.3 mg/d and 87% were taking β-blockers and a renin-angiotensin antagonist. A total of 26.1% of patients recruited had chronic kidney disease (CKD) stage 3a (eGFR, 45–60 mL/min/1.73 m^2^). The mean HbA1c was 7.9±3.8% (62.7±18.4 mmol/mol), with a mean duration of T2D of 8.7±6.3 years. Mean serum creatinine level was 87.3 mmol/L±13.6 mmol/L with urinary albumin/creatinine ratio 3.2±4.5 mg/mmol. There were no participant dropouts.

### Twenty-Four–Hour Urine Collection

**Figure 3.**
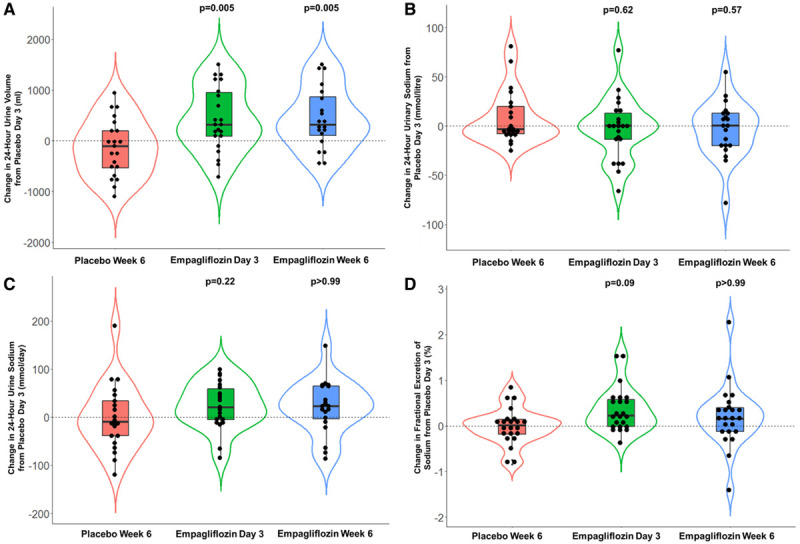
**Change in urine volume, urine sodium, and fractional excretion of sodium from placebo day 3.** Change in urine volume (**A**), urine sodium (**B** and **C**), and fractional excretion of sodium (**D**) from placebo day 3. *P* values refer to the mean difference between placebo week 6 and empagliflozin day 3 and week 6.

When compared with placebo, empagliflozin caused a significant increase in 24-hour urinary volume at both day 3 (mean difference, 535 mL [95% CI, 133–936]; *P*=0.005) and week 6 (mean difference, 545 mL [95% CI, 136–954]; *P*=0.005) when adjusted for treatment order, baseline 24-hour urine volume, and percentage change in loop diuretic dose (Table 2 and Figure 3A).

**Table 2.**
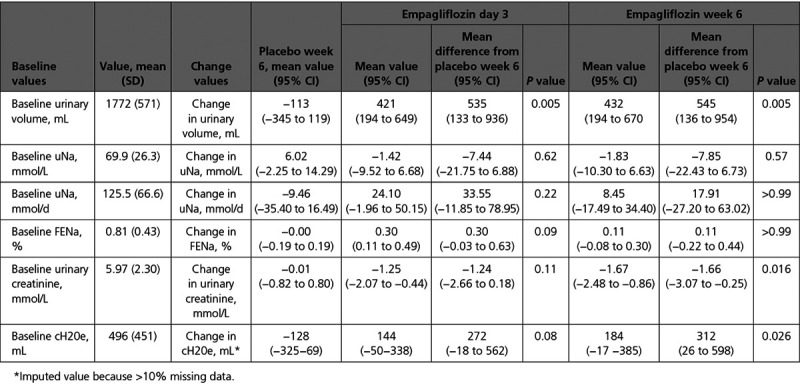
Change in 24-Hour Urine Volume, 24-Hour Urinary Sodium (uNa) mmol/L, mmol/d, and Fractional Excretion of Sodium (FENa %), 24-Hour Urinary Creatinine, and Electrolyte Free Water Clearance (cH20e) With Placebo and Empagliflozin at Day 3 and Week 6 Adjusted for Treatment Order, and Any Percentage Change in Loop Diuretic Dose With Baseline Values

There was no significant interaction between treatment order and the primary outcome (*P*=0.62). There was no significant difference in the change in 24-hour urinary volume from baseline dependent on the order of treatment (mean 246.3 mL in treatment order placebo/empagliflozin, mean 246.7 mL in treatment order empagliflozin/placebo, *P*>0.99), demonstrating no carryover effect in the primary outcome.

Empagliflozin did not cause a significant change in 24-hour urinary sodium measured in mmol/L (mean difference compared with placebo: day 3, −7.44 mmol/L [95% CI, −21.75 to 6.88]; *P*=0.62; week 6: −7.85 mmol/L [95% CI, −2.43 to 6.73]; *P*=0.57) or in mmol/d (mean difference: day 3, 33.6 mmol/d [95% CI, −11.8 to 79.0]; *P*=0.22; week 6: 17.9 mmol/d [95% CI, −27.2 to 63.0]; *P*>0.99; Figure 3B and 3C). Empagliflozin caused an increase in FENa during the 24-hour urine collections at day 3 (mean difference, 0.30% [95% CI, −0.03 to 0.63]; *P*=0.09) but this did not reach significance. There was no significant change in FENa as measured at week 6 (mean difference, 0.11% [95% CI, −0.22 to 0.44]; *P*>0.99) when compared with placebo (Figure 3D).

There was no significant difference in 24-hour urine volume, sodium, or FENa stratified by eGFR above or below 60 mL/min/1.73 m^2^ (interaction *P* values 0.43, 0.56, and 0.34, respectively).

Compared with placebo, empagliflozin caused a significant decrease in 24-hour urinary creatinine at week 6 (mean difference, −1.66 mmol/L [95% CI, −3.07 to −0.25]; *P*=0.016), with a nonsignificant decrease at day 3 (mean difference, −1.24 mmol/L [95% CI, −2.66 to 0.18]; *P*=0.11).

Empagliflozin caused a significant increase in electrolyte-free water clearance (cH20e) at week 6 (mean difference when compared with placebo 312 mL [95% CI, 26–598]; *P*=0.026), with a nonsignificant increase at day 3 (mean difference, 272 mL [95% CI, −18 to 562]; *P*=0.08).

Empagliflozin did not cause a significant change in urinary urea as measured in the 24-hour urine collection at both time points (day 3: mean difference, −21.83 mmol/L [CI, −58.99 to 39.19]; *P*>0.99; week 6: mean difference, −3.05 [CI, −40.84 to 34.74]; *P*>0.99).

### Renal Physiology Tests

**Figure 4.**
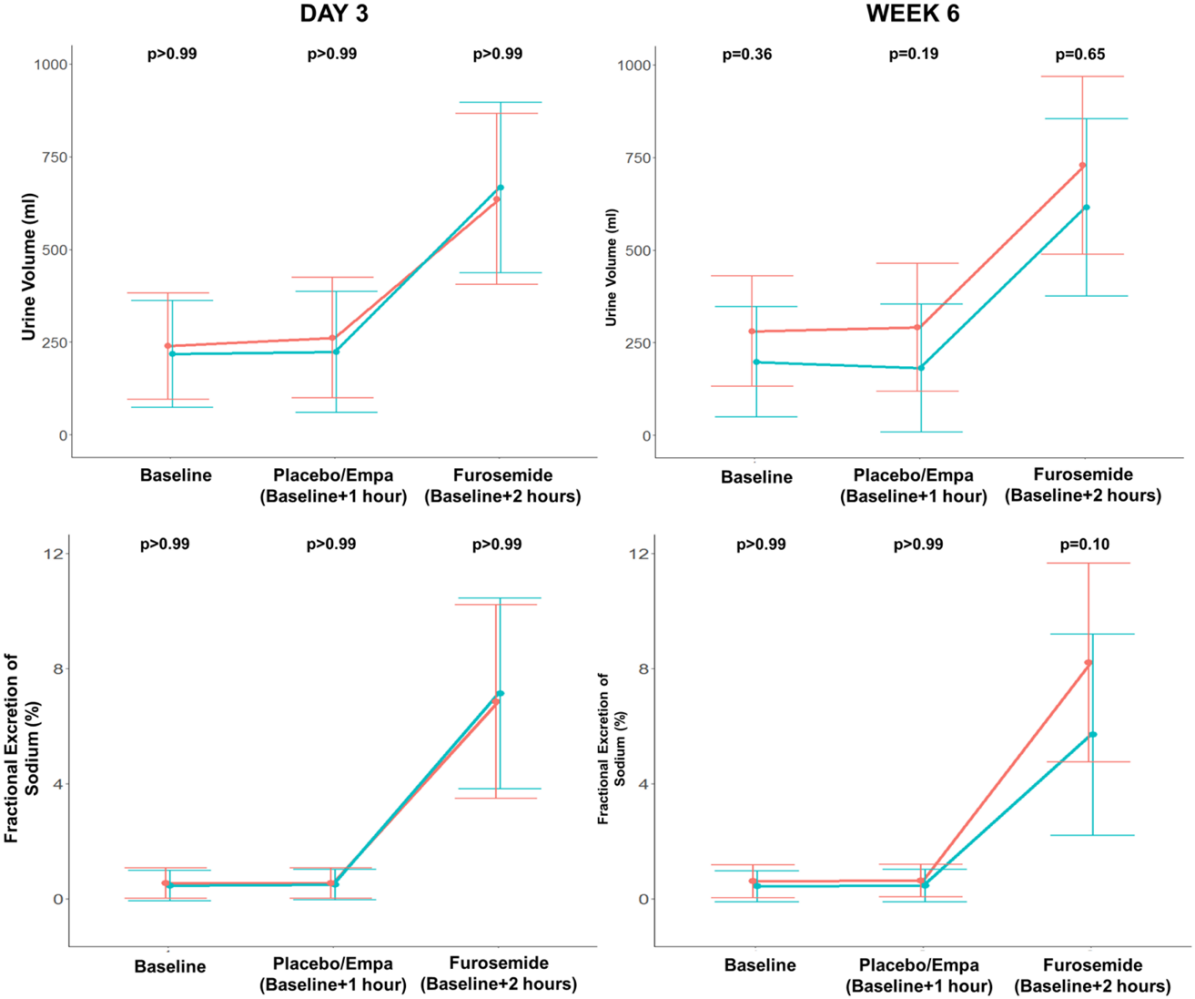
**Urine volume and fractional excretion of sodium during the renal physiologic test (RPT).** Mean and SD of urine volume (top row) and fractional excretion of sodium (bottom) at baseline and after administration of empagliflozin (Empa)/placebo and intravenous furosemide during the RPTs at day 3 and week 6. The red line represents the empagliflozin treatment arm, and the green line represents the placebo treatment arm.

RPT results are summarized in Figure 4 and Table II in the Data Supplement. In the RPTs, there were no significant differences in urinary volume, urine sodium, or FENa during the first hour (baseline measurement) at day 3 or week 6. In the hour after study drug administration, empagliflozin caused a nonsignificant increase in urine volume at week 6 (mean difference, 110 mL [95% CI, −26 to 246]; *P*=0.19). At week 6, patients taking empagliflozin had an increased FENa in the hour after administration of loop diuretic compared with when taking placebo, but again this did not reach statistical significance (mean difference, 2.51% [95% CI, −0.27 to 5.28]; *P*=0.10).

### Secondary Outcomes and Additional Measures

Empagliflozin did not cause any significant change in markers of renal function (serum creatinine, urine protein-to-creatinine ratio, urine albumin-to-creatinine ratio, or cystatin C) compared with placebo at day 3 or week 6 (Table III in the Data Supplement). A total of 12 (52.2%) participants saw a drop in eGFR category (ie, CKD stage 2 to CKD stage 3a by day 3 of empagliflozin compared with 7 in the placebo arm; *P*=0.13). However, this was transient, and renal function recovered in the majority of patients in the empagliflozin arm, with only 6 participants having a persistent reduction in CKD category versus 5 on placebo (*P*=0.73).

**Figure 5.**
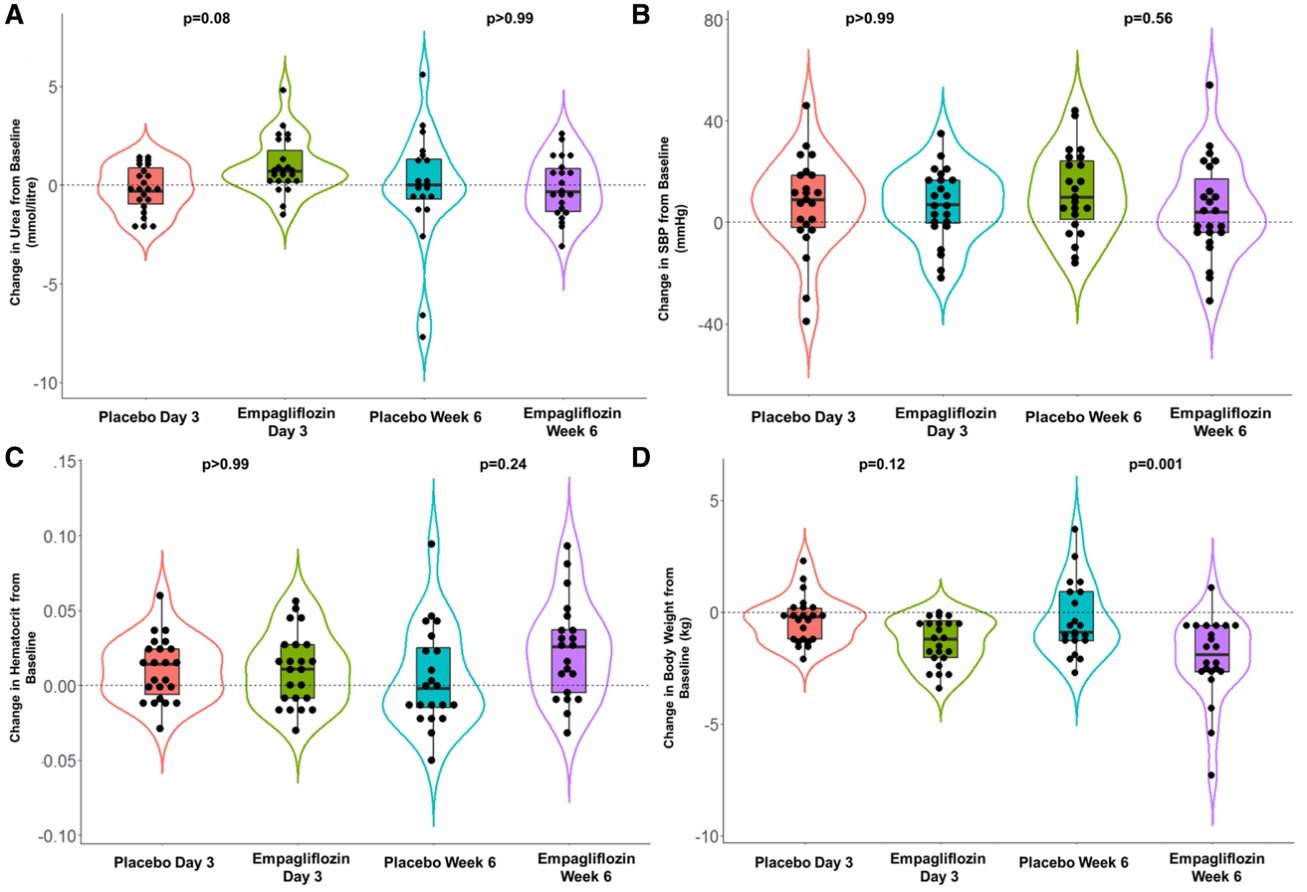
**Change in serum urea, systolic blood pressure, hematocrit, and body weight.** Change in serum urea (**A**), systolic blood pressure (SBP; **B**), hematocrit (**C**), and body weight (**D**). *P* values refer to the mean difference between empagliflozin and placebo at day 3 and week 6.

There were no significant differences in intravascular volume markers (serum urea [Table 3 and Figure 5A], systolic blood pressure [Figure 5B], and serum hematocrit [Figure 5C]). There was no significant change in NT-proBNP or serum β-hydroxybutyrate.

**Table 3.**
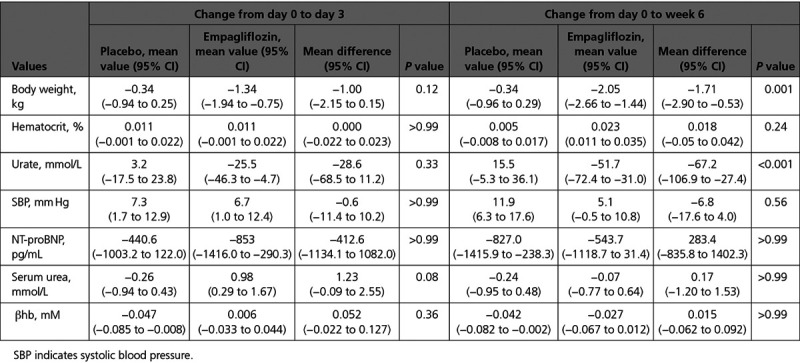
Change in Weight, Hematocrit, Urate, Systolic Blood Pressure, NT-proBNP (N-terminal pro–B-type natriuretic peptide), Serum Urea, and β-Hydroxybutyrate (βhb) With Placebo and Empagliflozin From Day 0 to Day 3 and Week 6 Adjusted for Treatment Order and Any Percentage Change in Loop Diuretic Dose

Empagliflozin caused significant weight loss compared with placebo at week 6 (day 3: mean difference, −1.00 kg [95% CI, −2.15 to 0.14]; *P*=0.12; week 6: mean difference, −1.71 kg [95% CI, −2.90 to −0.53]; *P*<0.001; Figure 5D). Empagliflozin also caused a significant reduction in serum urate by week 6 (day 3: mean difference, −28.6 [95% CI, −68.5 to 11.2]; *P*=0.33; week 6: mean difference, −67.2 [95% CI, −107.0 to −27.4]; *P*<0.001).

### Clinical Events

Clinical events are summarized in Table IV in the Data Supplement. Five patients required a 50% reduction of their furosemide dose while on the active treatment arm of empagliflozin by day 3 (1 patient had a 50% dose reduction from 80 mg to 40 mg once daily, 4 patients 50% reduction of 40 mg to 20 mg once daily) because of improvement in their clinical volume status. Of those patients who had their loop diuretic reduced, mean weight loss at day 3 was 2.22±1.19 kg. On discontinuation of empagliflozin, they needed to have their usual dose of loop diuretic reinstated because of weight gain or other clinical signs of increased congestion (within the blinded period). Two participants required their usual oral loop diuretic to be doubled after discontinuation of the active treatment arm with empagliflozin.

Two participants who had been on an unchanged dose of loop diuretic throughout the trial had hospital admissions with decompensated HF on discontinuation of empagliflozin. There were no episodes of hyponatremia or hypokalemia. There were no incidences of diabetic ketoacidosis or episodes of severe hypoglycemia (ie, episodes requiring assistance from another person to treat). Two patients experienced an acute decline in renal function as measured by a serum creatinine rise >26 mmol/L in 48 hours^13^ by day 3 of empagliflozin, which resolved by week 6.

## Discussion

In this randomized, controlled trial of the effects of SGLT2 inhibition in addition to loop diuretic in patients with HF with T2D, we found that within 6 weeks empagliflozin caused a significant increase in total urine volume without a significant increase in urinary sodium or FENa compared with placebo. We also found that empagliflozin caused a significant increase in electrolyte free water clearance. Empagliflozin also caused significant weight loss and overall changes in clinical status necessitating a reduction in furosemide dose. There were no significant changes in markers associated with intravascular volume change, including serum urea, hematocrit, or systolic blood pressure.

After DAPA-HF, SGLT2 inhibitors are increasingly being considered as an HF medication rather than as strictly antihyperglycemic therapy.^4^ The early benefit improvement in HF outcomes seen has raised the possibility that the osmotic diuresis caused by SGLT2 inhibitors might be one mechanism contributing to improved outcomes by improving ventricular loading conditions.^9^ Our study sheds light on this potential diuretic effect; our findings of a significant increase in urine volume without a significant increase in natriuresis with associated significant weight loss could be particularly beneficial in patients with HF in whom traditional strategies for reducing fluid overload—for example, combining loop and thiazide diuretics—can induce hyponatremia.^14^

We found that empagliflozin caused a significant increase in 24-hour urine volume but did not have a significant effect on urine volume or sodium during the RPTs immediately after a single dose. This has been reported in other studies, although we have now extended this finding to patients with HF with T2D on loop diuretic, including in those with eGFR 45 to 60 mL/min/1.73 m^2^. In a trial of 36 patients with T2D, canagliflozin caused an increase in total urine volume without any significant change in plasma volume at 12 weeks.^15^ In another small observational study in patients with CKD, dapagliflozin was associated with preferential reduction in extracellular water in comparison to furosemide.^16^ As a potential HF therapy, SGLT2 inhibitors could be frequently prescribed alongside loop diuretics, but there are little data on coadministration. A study of 42 healthy volunteers randomized to dapagliflozin, bumetanide, or combination therapy found that the combination of dapagliflozin and bumetanide caused a significant increase in urine volume, but not urinary sodium, compared with bumetanide alone.^17^ The authors also found that there was no significant change in natriuresis after the first dose of dapagliflozin, suggesting that there is an adaptive, synergistic increase in natriuresis over the first week.

Griffin et al^18^ recently demonstrated that empagliflozin monotherapy caused a modest natriuresis compared with placebo as measured by the FENa, which was significant at 3 hours after drug administration but not at 1.5 hours. They also reported a synergistic natriuretic effect when empagliflozin was given alongside intravenous loop diuretic. We did not note a significant change in FENa at 2 hours after empagliflozin or 1 hour after intravenous furosemide on the RPT days. It is possible that we may have seen an increase in FENa after a longer duration. Nevertheless, in the 24-hour urine collections, we also did not find an increase in natriuresis as demonstrated by mmol/L or mmol/d, and although there was a nonsignificant increase in FENa at day 3 caused by empagliflozin, this was absent by week 6. Our data are complementary to those of Griffin et al,^18^ and the differences in FENa between our study and that by Griffin et al^18^ may reflect the differing time points studied. In the study by Griffin et al,^18^ the largest effect of empagliflozin on FENa was beyond 3 hours at day 1, whereas at day 14, the increase in FENa with empagliflozin compared with placebo was attenuated. In a single-blind study, Blau et al^19^ found that although canagliflozin caused a significant increase in natriuresis at 24 hours, urine sodium had returned to baseline by 96 hours. A study by Opingari et al^20^ also did not demonstrate a significant change in FENa at 6 months in patients with T2D and established cardiovascular disease treated with empagliflozin when compared with placebo, supporting the theory that any natriuresis that might be caused by empagliflozin is perhaps transient and only present very early. This may be attributable to compensatory mechanisms that increase sodium reabsorption at other nephron sites.

FENa is a well-used calculation to quantify renal sodium handling, by normalizing sodium excretion by correcting for urinary and serum creatinine. After drug initiation, SGLT2 inhibitors are often associated with an initial increase in serum creatinine.^1,2,6,21^ Because creatinine filtration can be affected by SGLT2 initiation, FENa may be difficult to interpret in this context. We saw a nonsignificant increase in serum creatinine with empagliflozin that had resolved by week 6, a pattern well-described with SGLT2 inhibitors. Empagliflozin also caused a significant decrease in 24-hour urinary creatinine, which would also lead to an increase in calculated FENa.

Recent work from Hallow et al,^22^ using mathematical modeling in healthy participants after ingestion of SGLT2 inhibitor (dapagliflozin) or loop diuretic (bumetanide), suggested that SGLT2 inhibitors may provide a more selective reduction in interstitial fluid when compared with other conventional diuretics.^9^ The authors found that dapagliflozin caused an increase in electrolyte-free water clearance without a significant increase in total free water clearance, in contrast to bumetanide (which also caused an increase in total free water clearance). They suggested that this might result in a greater reduction in interstitial fluid volume compared with the intravascular compartment, possibly influenced by the peripheral sequestration of osmotically inactive sodium.^22^ In RECEDE-CHF, we also found a significant increase in electrolyte-free water clearance by week 6 in patients with HF, which if representative of a preferential loss of interstitial fluid would clearly be beneficial in HF.^9,19,22^ The hypothesis of SGLT2 inhibitor–induced preferential loss of fluid from the interstitial rather than the intravascular space remains speculative and cannot be directly inferred from our study.

We had some secondary findings that could be considered beneficial. Patients required a reduction in loop diuretic dose, which was also reported in the REFORM trial (Research Into the Effect of SGLT2 Inhibition on Left Ventricular Remodelling in Patients With Heart Failure and Diabetes Mellitus).^23^ After cessation of empagliflozin, some patients also noted increasing congestion requiring an increase in loop diuretic or had a HF hospitalization, although our trial was clearly not powered for clinical outcomes. In a post hoc analysis of DAPA-HF, the benefit of dapagliflozin was consistent irrespective of baseline diuretic use; however, in patients on a diuretic, adverse events related to volume depletion occurred more frequently with dapagliflozin compared with placebo.^24^ In addition, renal adverse events were less common with dapagliflozin compared with placebo in those not on a diuretic at baseline.^24^ These findings underscore the need for vigilance regarding volume status and judicious adjustment of loop diuretic doses when initiating an SGLT2 inhibitor in patients with HF.

The finding of a significant reduction in serum urate with empagliflozin has been documented previously; however, it is reassuring that we also observed this in patients with HF taking a regular loop diuretic.^25^ This may again provide evidence to favor use of SGLT2 inhibitors as an add-on therapy in patients with HF with fluid congestion rather than thiazide diuretics or other alternatives.

We did not find a significant change in NT-proBNP at 3 days or 6 weeks. Although this is perhaps counterintuitive given the overwhelming evidence of benefit of SGLT2 inhibitors in HF, this has actually also been reported in other trials including patients with chronic^26^ and acute HF.^27^ In both of these studies, SGLT2 inhibition led to a clinically important benefit despite the absence of a change in natriuretic peptide levels. This may in part be attributable to weight loss, which has been reported as causing an increase in NT-proBNP.^28,29^

Our findings may also have clinical implications in patients with decompensated HF and fluid overload. At this time, sequential nephron blockade with thiazide-like diuretics used in combination with loop diuretics is often used to overcome diuretic resistance in acute decompensated chronic HF.^30^ However, this strategy does not always work and is associated with the hazards of hypokalemia, hyponatremia, hypotension, and renal failure. We demonstrated that diuresis without significant natriuresis is quickly achieved by day 3. Potentially, this highlights the SGLT2 inhibitor as an alternative to the thiazide-like diuretics in patients who may have evidence of fluid retention resistance to loop diuretics alone.

Our study has some limitations. It was a small, single-center crossover study with participants who were predominantly male and with a midrange ejection fraction. The crossover design was a strength, with each patient acting as their own control in a blinded study. The dose investigated in this trial was 25 mg empagliflozin once daily, whereas in routine clinical practice the 10 mg dose is most commonly used; however, in EMPA-REG OUTCOME (BI 10773 [Empagliflozin] Cardiovascular Outcome Event Trial in Type 2 Diabetes Mellitus Patients), no difference in the primary outcome was reported between the 2 treatment groups of 10 mg and 25 mg.^1^ At the time we conceived the study (before the publication of EMPA-REG OUTCOME), we hypothesized that there might be a dose–response effect and therefore chose the 25 mg dose in this study. On the day of the 24-hour urine collection, we asked participants to restrict sodium intake to 2 g/d and fluid intake to 2 L/d; however, we could not enforce this because patients were not admitted to hospital but remained at home. Patients attended the RPT days after an overnight fast to minimize any variations in dietary glucose intake. There was no dietary restriction placed on participants while the 24-hour urine collection was performed, and any variation in glucose control may have modified the urinary responses. The effect of glucosuria from SGLT2 inhibition is well-described in the literature^18,20,31^ and as such we did not measure urine glucose in this study. Given that we demonstrated diuresis in the absence of significant natriuresis, it would have been interesting to identify whether there was an association between glucosuria and 24-hour urine volume. Because of time constraints during the RPT days, the patients were observed for only 2 hours after administration of investigational medical product (placebo or empagliflozin) and 1 hour after administration of intravenous furosemide. Any significant diuretic or natriuretic effect may have not been observed during this short time frame, and might have been seen with a longer duration. Assessment of plasma renin activity would also have been of interest in exploring intravascular volume, although this may have been influenced by high use of renin-angiotensin-aldosterone inhibitors in patients with HF. It is unclear whether the changes we saw at 6 weeks would persist for a longer time. One observational of study of patients with T2D, but without HF, reported a reduction in extracellular water at day 3, which had returned to baseline values by 3 months.^32^

### Conclusions

In the RECEDE-CHF trial, we found that in patients with T2DM and HF taking regular furosemide, 6 weeks of treatment with empagliflozin caused a significant increase in 24-hour urine volume without an increase in urinary sodium compared with placebo. Empagliflozin also caused a significant increase in electrolyte-free water clearance, significant weight loss, and reduced loop diuretic requirement. These findings, combined with a reduction in serum uric acid and no significant renal impairment or electrolyte disturbance, provide further insight into the mechanism of the diuretic effect of empagliflozin and suggest that the combination of loop diuretic and SGLT2 inhibition could have a beneficial role in HF.

## Acknowledgments

The authors thank the participants enrolled in the trial; staff members of the Division of Clinical and Molecular Medicine at the University of Dundee; and the team at the Clinical Research Center, University of Dundee, Ninewells Hospital, and Medical School, United Kingdom. Recruitment to this study was facilitated by SHARE, the Scottish Health Research Register, which is supported by NHS Research Scotland, all the Universities of Scotland, and the Chief Scientist’s Office of the Scottish Government. Dr N.A. Mordi was the principal investigator, trial manager, data manager, and trial statistician who wrote the manuscript. Dr I.R. Mordi contributed to the protocol design, statistical analysis, writing of the original manuscript, and critical revisions. Dr Singh, Dr McCrimmon, and Dr Struthers participated in review of the manuscript. Dr Lang conceived the original paper design, was the chief investigator, and reviewed the manuscript. All authors read and approved the final manuscript.

## Sources of Funding

The RECEDE-CHF trial (SGLT2 Inhibition in Combination With Diuretics in Heart Failure) was funded by the British Heart Foundation (BHF; grant 807998). Dr N.A. Mordi was a BHF-funded clinical research fellow. Dr I.R. Mordi is funded by a National Health Service Education for Scotland Postdoctoral Clinical Lectureship (PCL 17/07). Dr Singh was funded by a grant from the European Federation for the Study of Diabetes.

## Disclosures

Dr McCrimmon has received honoraria and speaker fees from Sanofi and honoraria from NovoNordisk and Lilly. Dr Struthers has received research support and lecture and consulting fees from Astra Zeneca. Dr Lang has received research support and consulting fees from Novartis; research support, lecture fees, and consulting fees from AstraZeneca; lecture fees from Merck Sharp & Dohme; and research support from Pfizer and Sanofi. The other authors report no conflicts.

## Supplemental Materials

Data Supplement Tables I– IV

## Supplementary Material



## References

[1] Zinman B Wanner C Lachin JM Fitchett D Bluhmki E Hantel S Mattheus M Devins T Johansen OE Woerle HJ; EMPA-REG OUTCOME Investigators Empagliflozin, cardiovascular outcomes, and mortality in type 2 diabetes. N Engl J Med. 2015;373:2117–2128. doi: 10.1056/NEJMoa1504720 26378978 10.1056/NEJMoa1504720

[2] Neal B Perkovic V Mahaffey KW de Zeeuw D Fulcher G Erondu N Shaw W Law G Desai M Matthews DR; CANVAS Program Collaborative Group Canagliflozin and cardiovascular and renal events in type 2 diabetes. N Engl J Med. 2017;377:644–657. doi: 10.1056/NEJMoa1611925 28605608 10.1056/NEJMoa1611925

[3] Wiviott SD Raz I Bonaca MP Mosenzon O Kato ET Cahn A Silverman MG Zelniker TA Kuder JF Murphy SA; DECLARE–TIMI 58 Investigators Dapagliflozin and cardiovascular outcomes in type 2 diabetes. N Engl J Med. 2019;380:347–357. doi: 10.1056/NEJMoa1812389 30415602 10.1056/NEJMoa1812389

[4] McMurray JJV Solomon SD Inzucchi SE Køber L Kosiborod MN Martinez FA Ponikowski P Sabatine MS Anand IS Bělohlávek J; DAPA-HF Trial Committees and Investigators Dapagliflozin in patients with heart failure and reduced ejection fraction. N Engl J Med. 2019;381:1995–2008. doi: 10.1056/NEJMoa1911303 31535829 10.1056/NEJMoa1911303

[5] McMurray J EMPA-REG: the “diuretic hypothesis.” J Diabetes Complications. 2016;30:3–4. doi: 10.1016/j.jdiacomp.2015.10.012 26597600 10.1016/j.jdiacomp.2015.10.012

[6] Verma S McMurray JJV Cherney DZI The metabolodiuretic promise of sodium-dependent glucose cotransporter 2 inhibition: the search for the sweet spot in heart failure. JAMA Cardiol. 2017;2:939–940. doi: 10.1001/jamacardio.2017.1891 28636701 10.1001/jamacardio.2017.1891

[7] Lytvyn Y Bjornstad P Udell JA Lovshin JA Cherney DZI Sodium glucose cotransporter-2 inhibition in heart failure: potential mechanisms, clinical applications, and summary of clinical trials. Circulation. 2017;136:1643–1658. doi: 10.1161/CIRCULATIONAHA.117.030012 29061576 10.1161/CIRCULATIONAHA.117.030012 PMC5846470

[8] Sattar N McLaren J Kristensen SL Preiss D McMurray JJ SGLT2 inhibition and cardiovascular events: why did EMPA-REG outcomes surprise and what were the likely mechanisms? Diabetologia. 2016;59:1333–1339. doi: 10.1007/s00125-016-3956-x 27112340 10.1007/s00125-016-3956-x PMC4901113

[9] Verma S McMurray JJV SGLT2 inhibitors and mechanisms of cardiovascular benefit: a state-of-the-art review. Diabetologia. 2018;61:2108–2117. doi: 10.1007/s00125-018-4670-7 30132036 10.1007/s00125-018-4670-7

[10] Richards M FDA approves new treatment for a type of heart failure. https://www.fda.gov/news-events/press-announcements/fda-approves-new-treatment-type-heart-failure. Published 2020. Accessed May 30, 2020

[11] Mordi NA Mordi IR Singh JS Baig F Choy AM McCrimmon RJ Struthers AD Lang CC Renal and cardiovascular effects of sodium-glucose cotransporter 2 (SGLT2) inhibition in combination with loop diuretics in diabetic patients with chronic heart failure (RECEDE-CHF): protocol for a randomised controlled double-blind cross-over trial. BMJ Open. 2017;7:e018097 doi: 10.1136/bmjopen-2017-018097 10.1136/bmjopen-2017-018097 PMC5652490 29042392

[12] Goldsmith SR Gilbertson DT Mackedanz SA Swan SK Renal effects of conivaptan, furosemide, and the combination in patients with chronic heart failure. J Card Fail. 2011;17:982–989. doi: 10.1016/j.cardfail.2011.08.012 22123359 10.1016/j.cardfail.2011.08.012

[13] Kidney Disease: Improving Global Outcomes (KDIGO) Acute Kidney Injury Work Group KDIGO clinical practice guideline for acute kidney injury. Kidney Int. 2012;2:1–138.

[14] Ravnan SL Ravnan MC Deedwania PC Pharmacotherapy in congestive heart failure: diuretic resistance and strategies to overcome resistance in patients with congestive heart failure. Congest Heart Fail. 2002;8:80–85. doi: 10.1111/j.1527-5299.2002.0758.x 11927781 10.1111/j.1527-5299.2002.0758.x

[15] Sha S Polidori D Heise T Natarajan J Farrell K Wang SS Sica D Rothenberg P Plum-Mörschel L Effect of the sodium glucose co-transporter 2 inhibitor canagliflozin on plasma volume in patients with type 2 diabetes mellitus. Diabetes Obes Metab. 2014;16:1087–1095. doi: 10.1111/dom.12322 24939043 10.1111/dom.12322

[16] Ohara K Masuda T Murakami T Imai T Yoshizawa H Nakagawa S Okada M Miki A Myoga A Sugase T Effects of the sodium-glucose cotransporter 2 inhibitor dapagliflozin on fluid distribution: a comparison study with furosemide and tolvaptan. Nephrology (Carlton). 2019;24:904–911. doi: 10.1111/nep.13552 30578654 10.1111/nep.13552

[17] Wilcox CS Shen W Boulton DW Leslie BR Griffen SC Interaction between the sodium-glucose-linked transporter 2 inhibitor dapagliflozin and the loop diuretic bumetanide in normal human subjects. J Am Heart Assoc. 2018;7:e007046 doi: 10.1161/JAHA.117.007046 29440005 10.1161/JAHA.117.007046 PMC5850181

[18] Griffin M Rao VS Ivey-Miranda J Fleming J Mahoney D Maulion C Suda N Siwakoti K Ahmad T Jacoby D Empagliflozin in heart failure: diuretic and cardio-renal effects. Circulation. 2020;142:1028–1039. doi: 10.1161/CIRCULATIONAHA.120.045691 32410463 10.1161/CIRCULATIONAHA.120.045691 PMC7521417

[19] Blau JE Bauman V Conway EM Piaggi P Walter MF Wright EC Bernstein S Courville AB Collins MT Rother KI Canagliflozin triggers the FGF23/1,25-dihydroxyvitamin D/PTH axis in healthy volunteers in a randomized crossover study. JCI Insight. 2018;3:e99123 doi: 10.1172/jci.insight.99123 10.1172/jci.insight.99123 PMC5931122 29669938

[20] Opingari E Verma S Connelly KA Mazer CD Teoh H Quan A Zuo F Pan Y Bhatt DL Zinman B The impact of empagliflozin on kidney injury molecule-1: a subanalysis of the Effects of Empagliflozin on Cardiac Structure, Function, and Circulating Biomarkers in Patients with Type 2 Diabetes CardioLink-6 trial. Nephrol Dial Transplant. 2020;35:895–897. doi: 10.1093/ndt/gfz294 32159783 10.1093/ndt/gfz294

[21] Wanner C EMPA-REG OUTCOME: the nephrologist’s point of view. Am J Cardiol. 2017;120:S59–S67. doi: 10.1016/j.amjcard.2017.05.012 28606346 10.1016/j.amjcard.2017.05.012

[22] Hallow KM Helmlinger G Greasley PJ McMurray JJV Boulton DW Why do SGLT2 inhibitors reduce heart failure hospitalization? A differential volume regulation hypothesis. Diabetes Obes Metab. 2018;20:479–487. doi: 10.1111/dom.13126 29024278 10.1111/dom.13126

[23] Singh JSS Mordi IR Vickneson K Fathi A Donnan PT Mohan M Choy AMJ Gandy S George J Khan F Dapagliflozin versus placebo on left ventricular remodeling in patients with diabetes and heart failure: the REFORM trial. Diabetes Care. 2020;43:1356–1359. doi: 10.2337/dc19-2187 32245746 10.2337/dc19-2187 PMC7245350

[24] Docherty KF Jhund PS Inzucchi SE Køber L Kosiborod MN Martinez FA Ponikowski P DeMets DL Sabatine MS Bengtsson O Effects of dapagliflozin in DAPA-HF according to background heart failure therapy. Eur Heart J. 2020;41:2379–2392. doi: 10.1093/eurheartj/ehaa183 32221582 10.1093/eurheartj/ehaa183 PMC7327533

[25] Zhao Y Xu L Tian D Xia P Zheng H Wang L Chen L Effects of sodium-glucose co-transporter 2 (SGLT2) inhibitors on serum uric acid level: a meta-analysis of randomized controlled trials. Diabetes Obes Metab. 2018;20:458–462. doi: 10.1111/dom.13101 28846182 10.1111/dom.13101

[26] Nassif ME Windsor SL Tang F Khariton Y Husain M Inzucchi SE McGuire DK Pitt B Scirica BM Austin B Dapagliflozin effects on biomarkers, symptoms, and functional status in patients with heart failure with reduced ejection fraction: the DEFINE-HF trial. Circulation. 2019;140:1463–1476. doi: 10.1161/CIRCULATIONAHA.119.042929 31524498 10.1161/CIRCULATIONAHA.119.042929

[27] Damman K Beusekamp JC Boorsma EM Swart HP Smilde TDJ Elvan A van Eck JWM Heerspink HJL Voors AA Randomized, double-blind, placebo-controlled, multicentre pilot study on the effects of empagliflozin on clinical outcomes in patients with acute decompensated heart failure (EMPA-RESPONSE-AHF). Eur J Heart Fail. 2020;22:713–722. doi: 10.1002/ejhf.1713 31912605 10.1002/ejhf.1713

[28] Fedele D Bicchiega V Collo A Barutta F Pistone E Gruden G Bruno G Short term variation in NTproBNP after lifestyle intervention in severe obesity. PLoS One. 2017;12:e0181212 doi: 10.1371/journal.pone.0181212 28704534 10.1371/journal.pone.0181212 PMC5509288

[29] Kistorp C Bliddal H Goetze JP Christensen R Faber J Cardiac natriuretic peptides in plasma increase after dietary induced weight loss in obesity. BMC Obes. 2014;1:24 doi: 10.1186/s40608-014-0024-2 26217511 10.1186/s40608-014-0024-2 PMC4511261

[30] Jentzer JC DeWald TA Hernandez AF Combination of loop diuretics with thiazide-type diuretics in heart failure. J Am Coll Cardiol. 2010;56:1527–1534. doi: 10.1016/j.jacc.2010.06.034 21029871 10.1016/j.jacc.2010.06.034

[31] Thomas MC Cherney DZI The actions of SGLT2 inhibitors on metabolism, renal function and blood pressure. Diabetologia. 2018;61:2098–2107. doi: 10.1007/s00125-018-4669-0 30132034 10.1007/s00125-018-4669-0

[32] Schork A Saynisch J Vosseler A Jaghutriz BA Heyne N Peter A Häring HU Stefan N Fritsche A Artunc F Effect of SGLT2 inhibitors on body composition, fluid status and renin-angiotensin-aldosterone system in type 2 diabetes: a prospective study using bioimpedance spectroscopy. Cardiovasc Diabetol. 2019;18:46 doi: 10.1186/s12933-019-0852-y 30953516 10.1186/s12933-019-0852-y PMC6451223

