# Supplementary material for: Renal and Cardiovascular Effects of SGLT2 Inhibition in Combination With Loop Diuretics in Patients With Type 2 Diabetes and Chronic Heart Failure: The RECEDE-CHF Trial
Source: Circulation. 2020 Aug 29;142(18):1713–24. doi: 10.1161/CIRCULATIONAHA.120.048739 (PMC7594536; doi:10.1161/CIRCULATIONAHA.120.048739)
Supplement: Supplementary file 1 [file cir-142-1713-s001.pdf]

# SUPPLEMENTAL MATERIAL

**SUPPLEMENTAL MATERIAL**

Supplemental Table I. Detailed Renal Physiology Test Day protocol.

Supplemental Table II. Full Renal Physiology Test Results.

Supplemental Table III. Additional Secondary Renal Outcomes for the RECEDE-CHF (change in Creatinine, uPCR, uACR and Cystatin C) with mean value, mean differences (95% CI) with placebo and empagliflozin at day 3 and week 6.

Supplemental Table IV. Adverse Events.

## Supplemental Table I: RENAL PHYSIOLOGY TEST DAY PROTOCOL

## 2 days pre-RPT: 2 gram sodium and 2L fluid intake

## 1-day pre-RPT: 24-hour urine collection begins

| Interval (minutes) | Urine | Measurement Period | Blood |
|---|---|---|---|
| - 30 | **Patient arrives fasted. 2 x cannula sited. 15mls/kg oral water load over 15 minutes** | | |
| - 15 | | | |
| 0 | | | |
| + 30 | Void urine. Water intake equal to urine volume. | | |
| + 60 | Void urine. Water intake equal to urine volume | | |
| + 90 | Void urine. Water intake equal to urine volume. | | |
| + 105 | | BASELINE (90-150 mins) | Bloods for U&Es & osmolality |
| + 120 | Void urine. Water intake equal to urine volume. Collect urine (urine B1) for volume, sodium, creatinine & osmolality. | | |
| + 135 | | | Bloods for U&Es & osmolality |
| + 150 | Void urine. Water intake equal to urine volume. Collect urine (urine B2) for volume, sodium, creatinine & osmolality | | |
| **INVESTIGATIONAL MEDICAL PRODUCT (EMPAGLIFLOZIN 25MG or PLACEBO) ADMINISTERED** | | | |
| + 165 | | EMPAGLIFLOZIN/PLACEBO (150-210 mins) | Bloods for U&Es & osmolality |
| + 180 | Void urine. Water intake equal to urine volume. Collect urine for volume, sodium, creatinine & osmolality | | |
| + 195 | | | Bloods for U&Es & osmolality |
| + 210 | Void urine. Water intake equal to urine volume. Collect urine for volume, sodium, creatinine & osmolality | | |
| **INTRAVENOUS FUROSEMIDE ADMINISTERED: HALF OF THE PATIENT'S SCREENING DOSE** | | | |
| + 225 | | FUROSEMIDE (210 – 270 mins) | Bloods for U&Es & osmolality |
| + 240 | Void urine. Water intake equal to urine volume. Collect | | |

| | | | |
|---|---|---|---|
| | urine for volume, sodium, creatinine & osmolality | | |
| + 255 | | | Bloods for U&Es & osmolality |
| + 270 | Collect urine for volume, sodium, creatinine & osmolality. | | |
| **END OF RPT** | | | |

**Supplemental Table II. Full Renal Physiology Test Results.**

| | Hour 1 (Baseline) | | | | Hour 2 (Empagliflozin or Placebo) | | | | Hour 3 (Intravenous furosemide) | | | |
|---|---|---|---|---|---|---|---|---|---|---|---|---|
| **Trial Period** | **Placebo (mean (SD))** | **Empa (mean (SD))** | **Mean Difference (95% CI)** | **p value** | **Placebo (mean (SD))** | **Empa (mean (SD))** | **Mean Difference (95% CI)** | **p value** | **Placebo (mean (SD))** | **Empa (mean (SD))** | **Mean Difference (95% CI)** | **p value** |
| **DAY 3** | | | | | | | | | | | | |
| **Urine Volume (ml)** | 218 (144) | 239 (144) | 20 (-93 to 134) | >0.99 | 223 (163) | 262 (163) | 38 (-91 to 168) | 0.99 | 667 (230) | 636 (230) | -31 (-212 to 151) | >0.99 |
| **Urine Sodium (mmol/l)** | 19.8 (17.7) | 17.2 (17.7) | -2.7 (-16.8 to 11.5) | >0.99 | 17.6 (15.8) | 17.1 (15.8) | -0.5 (-13.1 to 12.2) | >0.99 | 81.7 (20.6) | 76.0 (20.6) | -5.6 (-22.2 to 11.0) | >0.99 |
| **FENa (%)** | 0.45 (0.53) | 0.54 (0.53) | 0.09 (-0.33 t0 0.51) | >0.99 | 0.50 (0.53) | 0.55 (0.53) | 0.05 (-0.38 to 0.47) | >0.99 | 7.13 (3.31) | 6.85 (3.36) | -0.28 (-2.92 to 2.37) | >0.99 |

| | | | | | | | | | | | | |
|---|---|---|---|---|---|---|---|---|---|---|---|---|
| **WEEK 6** | | | | | | | | | | | | |
| **Urine Volume (ml)** | 198 (149) | 281 (149) | 84 (-35 to 203) | 0.36 | 181 (173) | 291 (173) | 110 (-26 to 246) | 0.19 | 615 (240) | 729 (240) | 114 (-76 to 305) | 0.65 |
| **Urine Sodium (mmol/l)** | 18.7 (18.2) | 16.5 (18.7) | -2.1 (-16.8 to 12.5) | >0.99 | 19.4 (17.3) | 16.2 (16.8) | -3.2 (-16.7 to 10.2) | >0.99 | 81.8 (21.6) | 75.4 (22.1) | -6.4 (-23.8 to 11.0) | >0.99 |
| **FENa (%)** | 0.43 (0.53) | 0.61 (0.57) | 0.18 (-0.26 to 0.62) | >0.99 | 0.46 (0.57) | 0.64 (0.57) | 0.18 (-0.28 to 0.63) | >0.99 | 5.70 (3.50) | 8.21 (3.45) | 2.51 (-0.27 to 5.28) | 0.10 |

FENa – Fractional Excretion of Sodium

**Supplemental Table III: Additional Secondary Renal Outcomes for the RECEDE-CHF (change in Creatinine, uPCR, uACR and Cystatin C) with mean value, mean differences (95% CI) with placebo and empagliflozin at day 3 and week 6**

| | Change from Day 0 to Day 3 | | | | Change from Day 0 to Week 6 | | | |
|---|---|---|---|---|---|---|---|---|
| | Placebo Mean (SD) | Empa Mean (SD) | Mean Difference (95% CI) | p value | Placebo Mean (SD) | Empa Mean (SD) | Mean Difference (95% CI) | p value |
| Creatinine (mmol/L) | -0.47 (-8.85 to 7.91) | 8.56 (0.18 to 16.94) | 9.03 (-7.08 to 25.13) | 0.80 | -10.81 (-19.20 to -2.44) | -0.44 (-8.82 to 7.94) | 10.38 (-5.73 to 26.48) | 0.51 |
| uPCR (mg/mmol) | -0.5 () | -3.04 (-7.06 to 0.97) | -3.00 (-10.71 to 4.72) | >0.99 | -3.05 (-7.05 to 0.97) | 2.26 (-1.75 to 6.28) | 5.31 (-2.41 to 13.02) | 0.40 |
| uACR (mg/mmol) | -0.38 (-2.26 to 1.49) | -1.15 (-3.02 to 0.72) | -0.77 (-4.31 to 2.87) | >0.99 | -1.10 (-2.98 to 0.77) | 1.18 (-0.69 to 3.05) | 2.29 (-1.3 to 5.89) | 0.54 |
| Cystatin C (ng/ml) | 4.72 (-104.24 to 113.68) | 148.35 (39.38 to 257.31) | 143.62 (-65.82 to 353.06) | 0.40 | 22.50 (-91.52 to 136.52) | 31.35 (-80.07 to 142.75) | 8.85 (-207.80 to 225.50) | >0.99 |

uPCR – urine protein/creatinine ratio; uACR – urine albumin/creatinine ratio

**Supplemental Table IV. Adverse Events.**

| Adverse Events | Empagliflozin n = 23 (%) | Placebo n = 23 (%) |
|---|---|---|
| Hospitalization with heart failure following discontinuation of investigational medicinal product | 2 (8.6) | 0 |
| Increase in loop diuretic dose following discontinuation of investigational medicinal product | 2 (8.6) | 0 |
| Rise in serum creatinine (by more than 26 mmol/L within 48 hours) | 2 (8.6) | 0 |
| Hypotension | 1 (4.3) | 0 |
| Gout | 1 (4.3) | 2 (8.6) |
